# Apoptosis-mediated endothelial toxicity but not direct calcification or functional changes in anti-calcification proteins defines pathogenic effects of calcium phosphate bions

**DOI:** 10.1038/srep27255

**Published:** 2016-06-02

**Authors:** Anton G. Kutikhin, Elena A. Velikanova, Rinat A. Mukhamadiyarov, Tatiana V. Glushkova, Vadim V. Borisov, Vera G. Matveeva, Larisa V. Antonova, Dmitriy E. Filip’ev, Alexey S. Golovkin, Daria K. Shishkova, Andrey Yu. Burago, Alexey V. Frolov, Viktor Yu. Dolgov, Olga S. Efimova, Anna N. Popova, Valentina Yu. Malysheva, Alexandr A. Vladimirov, Sergey A. Sozinov, Zinfer R. Ismagilov, Dmitriy M. Russakov, Alexander A. Lomzov, Dmitriy V. Pyshnyi, Anton K. Gutakovsky, Yuriy A. Zhivodkov, Evgeniy A. Demidov, Sergey E. Peltek, Viatcheslav F. Dolganyuk, Olga O. Babich, Evgeniy V. Grigoriev, Elena B. Brusina, Olga L. Barbarash, Arseniy E. Yuzhalin

**Affiliations:** 1 Research Institute for Complex Issues of Cardiovascular Diseases, Sosnovy Boulevard 6, Kemerovo, 650002, Russian Federation; 2 Institute of Coal Chemistry and Material Science under the Siberian Branch of the Russian Academy of Sciences, Sovietsky Avenue 18, Kemerovo, 650000, Russian Federation; 3 Kemerovo State University, Krasnaya Street 6, Kemerovo, 650043, Russian Federation; 4 Institute of Chemical Biology and Fundamental Medicine under the Siberian Branch of the Russian Academy of Sciences, Lavrentiev Avenue 8, Novosibirsk, 630090, Russian Federation; 5 Novosibirsk State University, Pirogova Street 2, Novosibirsk, 630090, Russian Federation; 6 Rzhanov Institute of Semiconductor Physics under the Siberian Branch of the Russian Academy of Sciences, Prospekt Lavrentieva 13, Novosibirsk, 630090, Russian Federation; 7 Institute of Cytology and Genetics under the Siberian Branch of the Russian Academy of Sciences, Prospekt Lavrentieva 10, Novosibirsk, 630090, Russian Federation; 8 Research Institute of Biotechnology, Kemerovo Institute of Food Science and Technology, Stroiteley Boulevard 47, Kemerovo, 650056, Russian Federation; 9 Kemerovo State Medical Academy, Voroshilova Street 22a, Kemerovo, 650029, Russian Federation; 10 Department of Oncology, Cancer Research UK and Medical Research Council Oxford Institute for Radiation Oncology, University of Oxford, Old Road Campus Research Building, Roosevelt Drive, OX3 7DQ, Oxford, United Kingdom

## Abstract

Calcium phosphate bions (CPB) are biomimetic mineralo-organic nanoparticles which represent a physiological mechanism regulating the function, transport and disposal of calcium and phosphorus in the human body. We hypothesised that CPB may be pathogenic entities and even a cause of cardiovascular calcification. Here we revealed that CPB isolated from calcified atherosclerotic plaques and artificially synthesised CPB are morphologically and chemically indistinguishable entities. Their formation is accelerated along with the increase in calcium salts-phosphates/serum concentration ratio. Experiments *in vitro* and *in vivo* showed that pathogenic effects of CPB are defined by apoptosis-mediated endothelial toxicity but not by direct tissue calcification or functional changes in anti-calcification proteins. Since the factors underlying the formation of CPB and their pathogenic mechanism closely resemble those responsible for atherosclerosis development, further research in this direction may help us to uncover triggers of this disease.

Calcium phosphate bions (CPB) were discovered as a cell culture contaminant by Kajander *et al*. more than 25 years ago, and results from their research group were published some years later^1^,^2^. These entities represent self-propagating particles of ≤500 nm in diameter and can be visualised using electron or atomic force microscopy^1^,^2^. Elemental analysis showed that CPB consist of carbon, nitrogen, oxygen, hydrogen, calcium, and phosphorus^1^,^2^. In addition, it was demonstrated that they adsorb several proteins^3^.

In Heiss *et al*.^4^ demonstrated that calcium and phosphates form CPB *in vitro* when reacting with fetuin-A, an anti-calcification protein^4^. Similar phenomenon was also reported by Price *et al*. who isolated a high molecular weight complex of calcium, phosphate, and fetuin-A from the serum of rats treated with etidronate, a bone mineralization inhibitor^5^,^6^,^7^. A further study by Raoult *et al*. confirmed the role of fetuin-A in the formation of CPB^8^. A group from Taiwan (Martel, Young, Wu, Wong, and colleagues) suggested CPB as a part of a physiological cycle that regulates the function, transport and disposal of calcium and phosphorus^9^,^10^,^11^,^12^,^13^,^14^,^15^. However, CPB can accumulate in the body upon an excess of precipitating calcium and phosphate ions or a failure of clearance mechanisms in the body fluid or cell milieu^13^. In addition, Kumon *et al*. suggested that CPB can be generated as by-products of chronic inflammation^16^. In a recent review, our group suggested that different entities, including hemoglobin-salt aggregates, products of lipid peroxidation, calcified membrane vesicles, and other inorganic substances may all possess nanoscale structure similar to CPB^17^.

A group from Taiwan demonstrated that CPB are indistinguishable when extracted from biological fluids^10^,^11^,^12^. However, CPB were also identified in calcified human tissues^18^,^19^,^20^,^21^,^22^,^23^,^24^,^25^,^26^,^27^,^28^, particularly arteries and heart valves^21^,^22^,^23^,^24^,^25^,^26^,^27^,^28^, but so far there were no attempts to compare tissue-derived CPB with the artificially synthesised ones. Moreover, there is still no experimental model that can decisively show whether CPB are the culprit of cardiovascular calcification or the innocent result of disturbed calcium and phosphorus homeostasis. Another major question is, if CPB are not the direct cause of calcification, do they induce other pathogenic effects promoting atherosclerosis? Theoretically, there can be at least three putative mechanisms of their pathogenicity: 1) endothelial toxicity; 2) direct tissue calcification; 3) functional changes in anti-calcification proteins.

We carried out an investigation with the two main aims: 1) to demonstrate identity or difference between CPB isolated from calcified tissues and artificially synthesised CPB; 2) to determine the role of CPB in cardiovascular calcification.

## Results

### Natural and artificial calcium phosphate bions are morphologically and chemically indistinguishable entities

**Figure 1:**
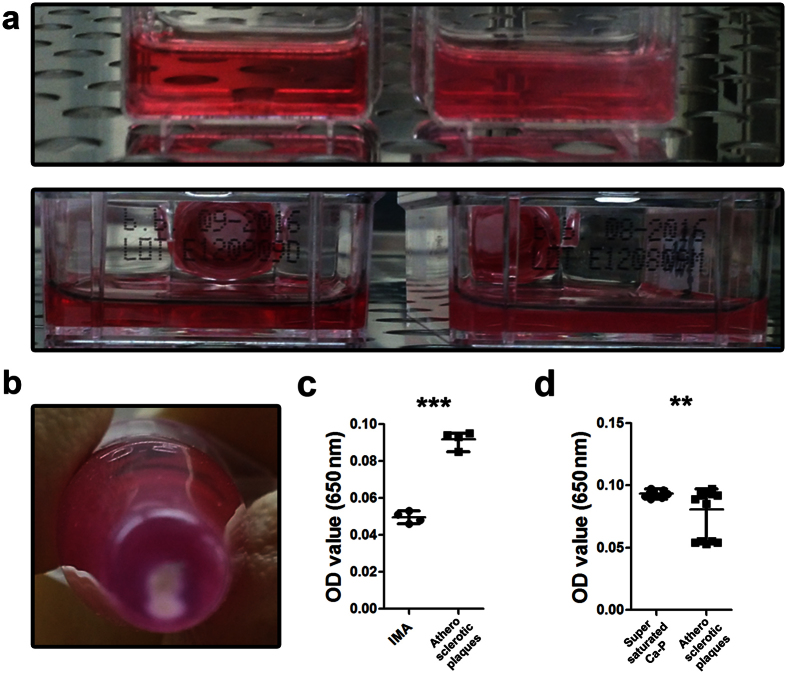
Natural calcium phosphate bions (CPB) may be cultured from calcified but not from non-calcified tissue. (**a**)An increased turbidity of the culture medium was observed in the flasks where the extracts of the calcified atherosclerotic plaques were added (right); however, the medium in the flasks where the extracts of internal mammary artery were added remained clear (left). (**b**)A white precipitate was found at the bottom of the tubes filled with the culture medium containing the extract of the calcified atherosclerotic plaque. (**c**) An increase in the optical density confirms the presence of certain particles in the culture medium with the extracts of the calcified atherosclerotic plaques (right, n = 4) but not with the extracts of internal mammary artery (left, n = 4). (**d**)Addition of the plaque extracts from eleven out of sixteen patients to the culture medium led to an increase in the optical density indicating formation of natural CPB (right, n = 16); optical density also increased indicating formation of artificial CPB when we added the supersaturated solution of CaCl_2_/Na_2_HPO_4_*12H_2_O to the culture medium (left, n = 16). All experiments were performed in triplicate. Each dot represents a biological replicate. Values are represented as mean with range. **P < 0.01, ***P < 0.001, two-tailed Student’s t-test.

**Figure 2:**
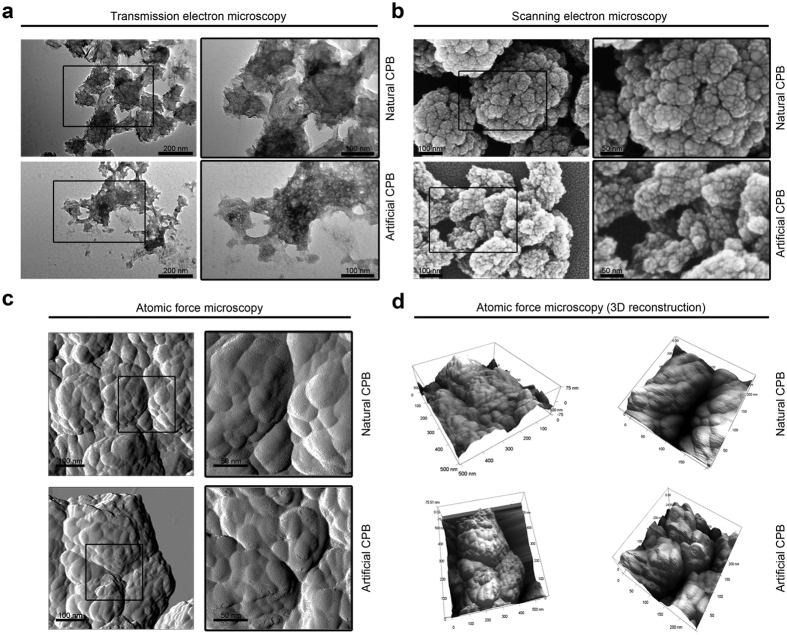
Both natural and artificial CPB have an indistinguishable morphology. (**a**) Transmission electron microscopy images of natural (top) and artificial (bottom) CPB. (**b**) Scanning electron microscopy images of natural (top) and artificial (bottom) CPB. (**c**) Atomic force microscopy images of natural (top) and artificial (bottom) CPB. (**d**) Three-dimensional atomic force microscopy images of natural (top) and artificial (bottom) CPB. All these images (**a**–**d**) confirm that both these entities are 100–500 nm spherical particles with a sponge-like structure and are morphologically indistinguishable. All experiments were performed in triplicate.

We first attempted to culture CPB from calcified atherosclerotic plaques (hereinafter: natural CPB) by adding the plaque extracts to the culture medium. We used the extracts of internal mammary artery as a negative control since it undergoes atherosclerosis extremely rarely and therefore confidently has no excessive concentrations of calcium or phosphorus^29^,^30^. Then, we cultured artificially synthesised (hereinafter: artificial) CPB by adding supersaturated solutions of CaCl_2_ and Na_2_HPO_4_*12H_2_O to the culture medium. After six weeks of culture, the medium in the flasks with either plaque extracts or supersaturated chemical solutions became turbid (Fig. 1a). A white precipitate was observed at the bottom of the respective tubes (Fig. 1b). Measurement of the optical density (OD) confirmed the formation of certain particles in the medium (Fig. 1c). However, no turbidity or OD increase were detected in the flasks or tubes with internal mammary artery extracts added to the medium (Fig. 1a,c). Extracts from the majority of patients (11/16) yielded precipitates and a significant OD increase, indicating CPB presence (Fig. 1d). Electron and atomic force microscopy revealed 100–500 nm spherical particles with a sponge-like structure (Fig. 2a–d), and confirmed that both natural and artificial CPB are morphologically indistinguishable.

**Figure 3:**
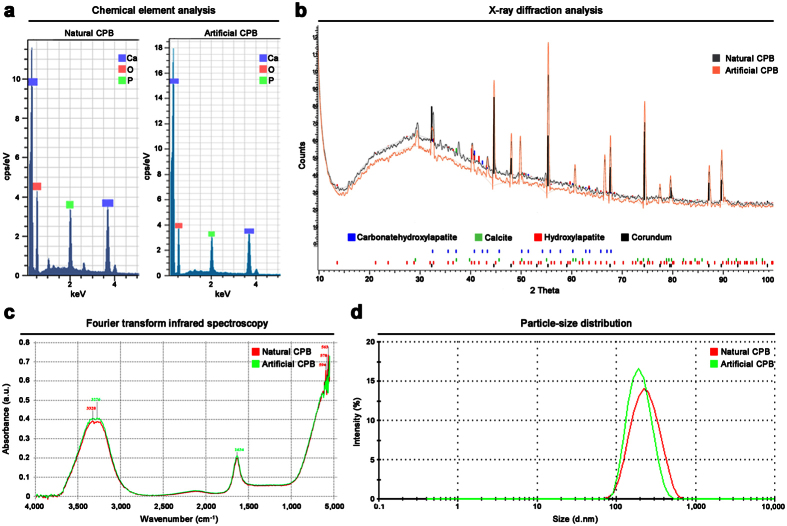
Both natural and artificial CPB have indistinguishable mineral pattern and particle-size distribution. (**a**) Energy-dispersive X-ray spectroscopy of natural (left) and artificial (right) CPB shows that they both consist at least of oxygen, calcium, and phosphorus (carbon cannot be seen here due to a carbon correction). (**b**) X-ray diffraction analysis identifies that both natural (grey) and artificial (pink) CPB are composed of hydroxylapatite [Ca_10_(PO_4_)_6_(OH)_2_], carbonate-hydroxylapatite [Ca_10_(PO_4_)_3_(CO_3_)_3_(OH)_2_], and calcite (CaCO_3_); corundum peaks are due to the plate where CPB were dried. (**c**) Fourier transform infrared spectroscopy reveals the similar peaks specific for the phosphate groups (550–600 cm^−1^) in both natural (red) and artificial (green) CPB; other peaks are due to ddH_2_O. (**d**) Dynamic light scattering measurements demonstrate that both natural (red) and artificial (green) CPB have a similar particle-size distribution curve, with a diameter range of 100–500 nm and mean diameter of around 200 nm. All experiments were performed in triplicate.

With the aim to compare a mineral pattern of the natural and artificial CPB, we performed an elemental analysis, Fourier transform infrared spectroscopy (FTIR), and an X-ray diffraction (XRD) analysis. All these techniques demonstrated that both natural and artificial CPB consist of carbon, oxygen, hydrogen, calcium, and phosphorus which form hydroxylapatite [Ca_10_(PO_4_)_6_(OH)_2_], carbonate-hydroxylapatite [Ca_10_(PO_4_)_3_(CO_3_)_3_(OH)_2_], and calcite (CaCO_3_) (Fig. 3a–c). Dynamic light scattering (DLS) measurements showed that the particle-size distribution of natural and artificial CPB is also nearly identical, with a diameter range of 100–500 nm and mean diameter of around 200 nm (Fig. 3d).

**Figure 4:**
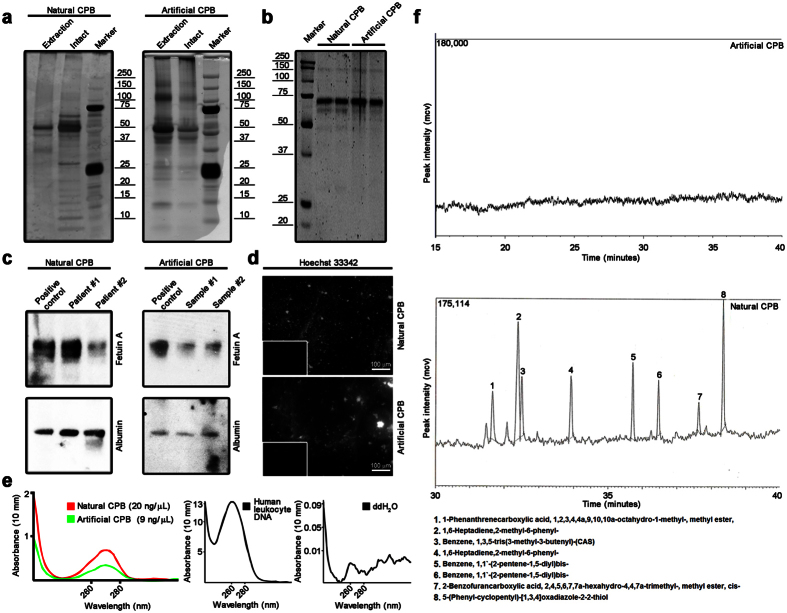
Both natural and artificial CPB have an indistinguishable organic pattern. (**a**) Sodium dodecyl sulphate-polyacrylamide gel electrophoresis following silver staining show that both natural (left) and artificial (right) CPB have a similar protein profile with at least three prominent bands at approximately 25 kDa, 50–58 kDa, and 65 kDa which may correspond to apolipoprotein A, fetuin-A, and albumin, respectively. (**b**) Decalcification significantly reduces the number of proteins in both natural (left) and artificial (right) CPB. (**c**) Western blotting confirms that both natural (left) and artificial (right) CPB contain fetuin A (top) and albumin (bottom). (**d**) Hoechst 33342 staining reveals double-stranded DNA adsorbed to both natural (top) and artificial (bottom) CPB; bottom inserts represent negative control staining of an empty slide. (**e**) Spectrophotometry shows peaks at a wavelength of 260/280 nm characteristic for dsDNA; human leukocyte DNA and ddH_2_O are provided as positive and negative controls, respectively. (**f**) Gas chromatography-mass spectrometry identifies a few residual lipids in natural (bottom) but not in artificial (top) CPB. All experiments were performed in triplicate.

Then, we compared an organic pattern of natural and artificial CPB. Sodium dodecyl sulphate-polyacrylamide gel electrophoresis (SDS-PAGE) following silver staining showed that they both have a similar protein profile with at least three prominent bands at approximately 25 kDa, 50–58 kDa, and 65 kDa which may correspond to apolipoprotein A, fetuin-A, and albumin, respectively (Fig. 4a). Decalcification of CPB using HCl or ethylenediaminetetraacetic acid (EDTA) disodium salt dihydrate significantly reduced the number of proteins but the albumin band was still prominent (Fig. 4b). These results were confirmed by Western blotting (WB) for fetuin-A and albumin (Fig. 4c). Both natural and artificial CPB were positively stained by Hoechst 33342 that indicated adsorption of double-stranded DNA (Fig. 4d). Spectrophotometry confirmed the presence of dsDNA in both types of CPB (Fig. 4e). Having performed a lipidomic profiling by gas chromatography-mass spectrometry (GC-MS), we found trace amounts of lipids in natural CPB but no lipids in artificial CPB (Fig. 4f).

### Calcium phosphate bions form under supersaturation of calcium salts and phosphates

**Figure 5:**
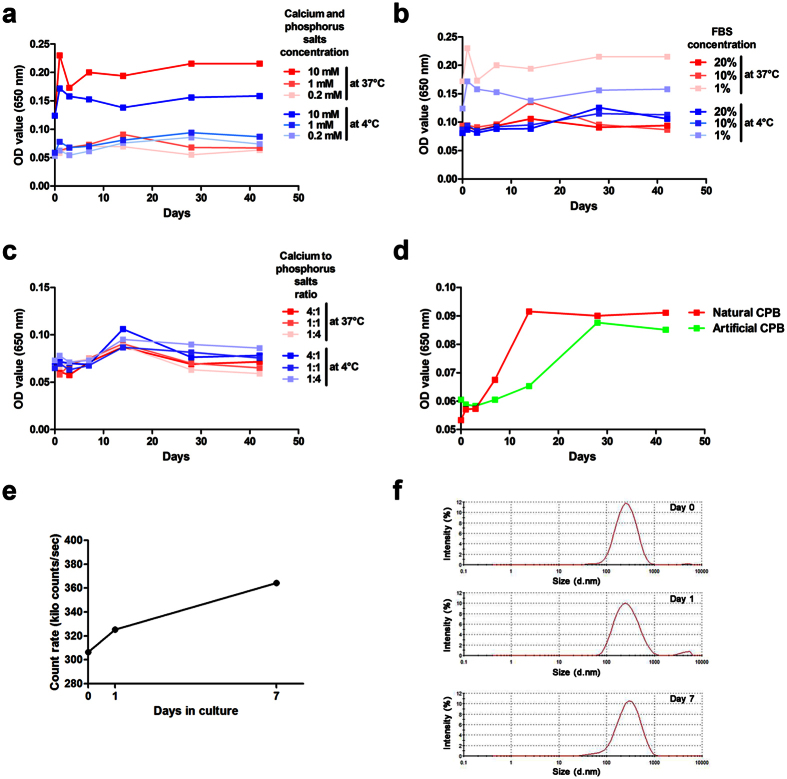
Calcium salts, phosphates, and serum determine the formation of the self-propagating CPB. (**a**) An increase in the concentration of calcium salts and phosphates promotes the formation of CPB both at 4 and 37 °C. (**b**) A decrease in the fetal bovine serum concentration also enhances this process both at 4 and 37 °C. (**c**) The ratio of calcium salts to phosphates does not affect the formation of CPB both at 4 and 37 °C. (**d**) Both natural (red) and artificial (green) CPB are able to self-propagate. (**e**) DLS particle concentration analysis of CPB cultured at 4 °C for 7 days. (**f**) DLS particle size distribution analysis of CPB cultured at 4 °C for 7 days. All experiments were performed in triplicate.

To define conditions determining the formation of CPB, we replicated a dual inhibition-seeding assay designed by the group from Taiwan^8^,^9^,^10^. An increase in the concentration of calcium salts and phosphates promoted the propagation of CPB regardless of the fetal bovine serum (FBS) concentration both under mammalian cell culture conditions and at 4 °C (Fig. 5a). Not surprisingly, a decrease in the FBS concentration also enhanced the formation of CPB regardless of the concentration of calcium salts and phosphates both under mammalian cell culture conditions and at 4 °C (Fig. 5b). The ratio of calcium salts to phosphates did not affect the formation of CPB regardless of the FBS concentration (Fig. 5c).

Finally, we detected a self-propagation ability of the both natural and artificial CPB cultured at 4 °C for over 40 days (Fig. 5d). Because turbidity measurements have limited quantitative power, we then reinforced our results with time resolved DLS analysis of particle concentration during the culture of natural CPB. We demonstrated a gradual increase of CPB concentration at days 1 and 7 (Fig. 5e), while particle size distribution remained unchanged (Fig. 5f).

Since all our experiments demonstrated that natural and artificial CPB are indistinguishable entities, we used artificial CPB for the further experiments.

### Endothelial toxicity defines pathogenic effects of calcium phosphate bions

**Figure 6:**
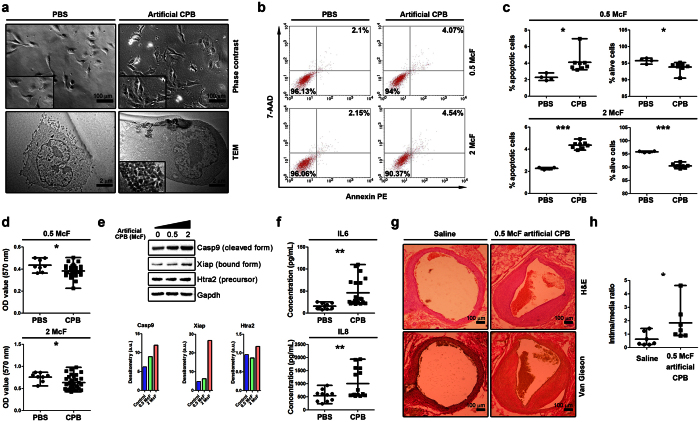
CPB are able to cause endothelial toxicity. (**a**) Phase contrast microscopy shows the presence of CPB amongst exposed EA.hy 926 cells (upper right corner) but not in the control culture (upper left corner); transmission electron microscopy confirms internalisation of CPB by exposed EA.hy 926 cells (lower right corner); the control culture is free of CPB (lower left corner). (**b**) Annexin V/7-aminoactinomycin D assay demonstrates that CPB are able to enhance apoptosis and reduce the viability of EA.hy 926 cells (a flow cytometry plot). (**c**) Annexin V/7-aminoactinomycin D assay demonstrates that CPB are able to enhance apoptosis and reduce the viability of EA.hy 926 cells (a graph comparison, n = 8 for the cultures exposed to CPB and n = 4 for the control cultures). (**d**) MTT assay confirms lower viability of EA.hy 926 cells exposed to CPB (right, n = 32) in comparison with the control cells (left, n = 8). (**e**) Western blot analysis of indicated proteins after treatment of endothelial cells with the ascending concentration of artificial CPB (0, 0.5, and 2 McF). Gapdh was used as a loading control. Densitometry analysis of bands normalised to the loading control is provided below. (**f**) Enzyme-linked immunosorbent assay shows that exposure to CPB promotes the production of IL-6 and IL-8 by EA.hy 926 cells (n = 20 for the cultures exposed to CPB and n = 10 for the control cultures). (**g**) H&E (top) and van Gieson (bottom) staining shows a significant intimal hyperplasia in abdominal aortas of rats with intravenously administered CPB (right) but not in control rats (left). (**h**) Intima/media ratio is higher in abdominal aortas of rats with intravenously administered CPB (right, n = 7) compared to control rats (left, n = 7). All experiments were performed in triplicate. Each dot represents a biological replicate. Values are represented as mean with range. *P < 0.05, **P < 0.01, ***P < 0.001, two-tailed Student’s t-test.

As we could observe CPB in the cell cultures using phase contrast microscopy, we attempted to detect the internalisation of CPB by EA.hy 926 human endothelial cell line, and successfully identified it using thin-section transmission electron microscopy (Fig. 6a).

We then investigated the ability of CPB to induce cytotoxic effects, i.e. promote apoptosis and decrease cell viability. Annexin V/7-aminoactinomycin D assay revealed that the percentage of cells undergoing late apoptosis/necrosis was significantly higher whilst the percentage of viable cells was significantly lower in EA.hy 926 cultures exposed to CPB for 24 h compared to the control cultures (Fig. 6b,c); the latter result was also confirmed by 3-(4,5-dimethylthiazol-2-yl)-2,5-diphenyltetrazolium bromide (MTT) assay (Fig. 6d). In order to provide a mechanistic insight on CPB-driven apoptosis, we analysed levels of apoptosis-related proteins in endothelial cells exposed to CPB for 18 h. We identified that CPB-induced apoptosis was mediated through a caspase-dependent pathway with an elevated level of cleaved caspase 9 in a dose dependent manner (Fig. 6e). We also observed an increased concentration of caspase-bound X-linked inhibitor of apoptosis protein (XIAP) in CPB-treated cells. Transiently increased levels of XIAP inhibitor HtrA2/Omi were only observed at the highest concentration of CPB (Fig. 6e).

Furthermore, we used an enzyme-linked immunosorbent assay (ELISA) to evaluate the cell culture supernatant for the levels of certain pro-atherosclerotic cytokines and cell adhesion molecules (IL-1α, -4, -6, -8, sCD40L, GM-CSF, sE-selectin, sP-selectin, sICAM-1, IFN-γ, and TNF-α) upon the exposure to CPB for 24 h. The levels of IL-6 and IL-8 were significantly higher in the supernatant from EA.hy 926 cultures exposed to CPB compared to the control cultures (Fig. 6f). In addition, we did not find any significant cytotoxicity or alterations of a secretory profile in primary cultures of the human dermal fibroblasts (data not shown).

Finally, we assessed whether CPB are able to cause intimal hyperplasia *in vivo*. In the experimental model of rat aorta angioplasty with a coronary angioplasty balloon catheter, we observed intimal hyperplasia in six out of seven rats with intravenously administered CPB but only in three out of seven control rats. Moreover, we detected significant concentric or eccentric intimal hyperplasia in abdominal aortas of three rats with intravenously administered CPB but in none of control rats (Fig. 6g). An intima/media ratio (IMR) was also significantly higher in rats with intravenously administered CPB in comparison with control rats (Fig. 6h).

### Calcium phosphate bions abrogate hedgehog signaling in endothelial cells

**Figure 7:**
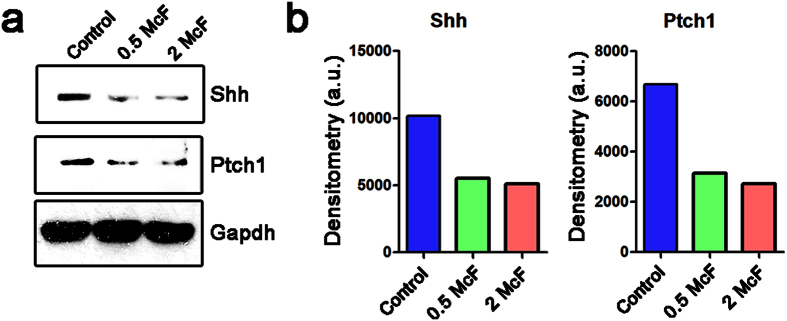
Calcium phosphate bions abrogate hedgehog signaling in endothelial cells. (**a**) Western blot analysis of Shh and Patch1 in 2H-11 cell lysates after treatment with the ascending concentration of artificial CPB (0, 0.5, and 2 McF). Gapdh was used as a loading control. (**b**) Densitometry analysis of bands as in (**a**) normalised to the loading control.

Hedgehog (HH) signaling is implicated in pathological calcification of organs and bone disease^31^. Recent evidence indicated a decreased activity of HH pathway in human carotid atherosclerotic plaques, as opposed to unaffected vessels^32^. To better characterise the impact of CPB on vascular behaviour, we assessed HH signaling in 2H-11 murine endothelial cell line exposed to CPB for 18 h. Intracellular protein levels of both Sonic Hedgehog (Shh) and its corresponding receptor, Patched1 (Ptch1), exhibited a twofold reduction after exposure to either 0.5 McF or 2 McF CPB (Fig. 7a,b). These data suggest that the effects of CPB on endothelial biology involve HH signaling.

### Direct tissue calcification and functional changes in anti-calcification proteins do not define pathogenic effects of calcium phosphate bions

**Figure 8:**
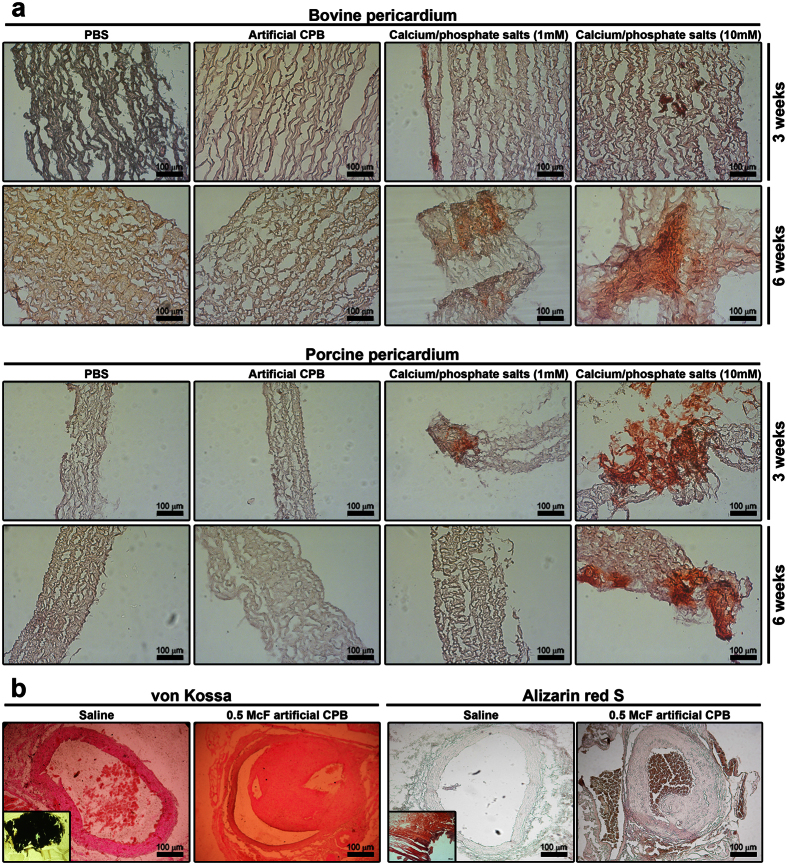
CPB are not able to cause direct tissue calcification. (**a**) Alizarin red S staining reveals calcium deposits in the bovine (top) and porcine (bottom) pericardia exposed to supersaturated solutions of CaCl_2_ and Na_2_HPO_4_*12H_2_O (right) but not in those exposed to phosphate buffered saline (control) or CPB (left). (**b**) Alizarin red S (right) and von Kossa (left) staining shows no calcium deposits in abdominal aortas of rats with intravenously administered CPB and control rats; bottom inserts represent positive control staining of human calcified bioprosthetic heart valves. All experiments were performed in triplicate.

To test whether CPB are able to induce direct calcification, we initially performed the experiments using our *in vitro* model of pericardial tissue calcification, where dissected bovine or porcine pericardia were cultured in either supersaturated salt solution or CPB. We did not detect any calcification either in bovine or in porcine pericardia exposed to CPB; however, exposure to CaCl_2_ and Na_2_HPO_4_*12H_2_O in equal concentrations of 1 mM or 10 mM did cause a significant calcification of the pericardia (Fig. 8a). To confirm these results, we performed von Kossa and alizarin red S staining of tissue sections from abdominal aortas of rats treated with CPB (Fig. 8b). Again, we could not observe any calcified areas.

**Figure 9:**
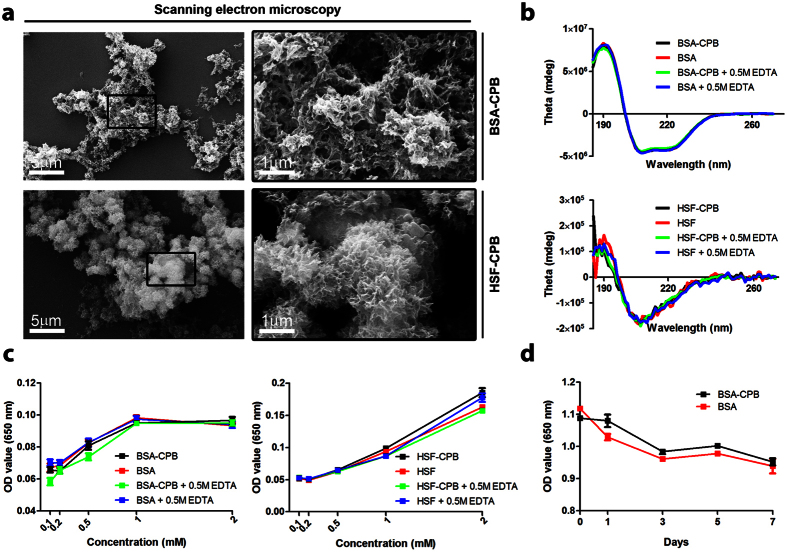
CPB are not able to cause structural or functional changes in anti-calcification proteins. (**a**) Scanning electron microscopy demonstrates a similarity of CPB containing the serum proteins with those containing only BSA (top) or HSF (bottom). (**b**) Circular dichroism spectra of the BSA and HSF incubated with either BSA-CPB solution, HSF-CPB solution, or ddH_2_O with or without further decalcification are similar and therefore show no structural consequences of the exposure to CPB. (**c**) Calcification propensity assay of the BSA and HSF incubated with either BSA-CPB solution, HSF-CPB solution, or ddH_2_O with or without further decalcification reveals no functional consequences of the exposure to CPB. (**d**) Precipitation assay shows no reduction of the BSA concentration during the incubation with BSA-CPB solution. All experiments were performed in triplicate.

To examine whether CPB are able to cause functional changes in anti-calcification proteins, we performed a number of experiments on CPB cultured exclusively with bovine serum albumin (BSA-CPB) or human serum fetuin-A (HSF-CPB). Electron microscopy revealed that morphology of the BSA-CPB and HSF-CPB was similar to serum-derived CPB (Fig. 9a). For the assessment of conformational changes in protein structure, we measured circular dichroism spectra of BSA and HSF incubated with either BSA-CPB solution, HSF-CPB solution, or ddH_2_O with or without further decalcification. No significant differences between the spectra were detected (Fig. 9b). We then investigated the anti-calcification function of BSA and HSF. As expected, calcification propensity assay did not show any significant differences between the samples (Fig. 9c). Finally, we tested the ability of CPB to precipitate BSA from solution. No significant differences were detected between the samples (Fig. 9d).

## Discussion

To the best of our knowledge, we are the first who performed a comparative analysis of natural and artificial CPB. Previously, it was shown that CPB isolated from a commercially available FBS and serum of healthy subjects are indistinguishable^10^,^11^,^12^. Recently, Wong *et al*. compared CPB from human kidney tissues explanted from patients with end-stage chronic kidney disease or renal cancer to the artificial ones, concluding that they are indistinguishable^33^. However, Wong *et al*. did not attempt to isolate tissue-derived CPB, and therefore this study was purely observational and limited to electron microscopy and elemental analysis^33^. Here, we demonstrated that natural and artificial CPB are morphologically and chemically indistinguishable entities. Albeit we identified certain minor differences in the lipidomic profile, they were likely to be caused by residual lipids from the atherosclerotic plaque extract since no clear lipid peaks were observed in artificial CPB. These residual lipids are unlikely to play major biological roles, and therefore we do not anticipate lipids to be a characteristic of natural CPB which can distinguish them from the artificial ones.

We isolated CPB from eleven out of sixteen atherosclerotic plaques but not from the extracts of internal mammary artery, which extremely rarely undergoes atherosclerosis with only a few cases reported in the most recent literature^29^,^30^. Therefore, we suggest that it was indeed a supersaturated calcium salts and phosphates condition that led to the formation of CPB in atherosclerotic vessels. Our further experiments confirmed the hypothesis of the group from Taiwan that calcium salts and phosphates enhance the seeding of CPB whilst serum, albumin, and fetuin-A inhibit this process^10^,^11^,^12^,^13^. Therefore, it is the calcium salts-phosphates/serum concentration ratio that crucially determines the formation of CPB. In other words, it is hypercalcemia/hyperphosphatemia that drives this process in the human blood. We further attempted to find out whether calcium salts or phosphates play the main role in the formation of CPB; however, the ratio of calcium salts to phosphates did not affect this process regardless of serum concentration.

We confirmed the ability of CPB to self-propagate using turbidimetry and time resolved DLS analysis. It should be noticed that the turbidimetry is not an accurate technique for the detection of a self-propagation since turbidity strongly depends on the particle size distribution, as large particles scatter significantly more light compared to small ones. In addition, turbidimetry cannot distinguish the true self-propagation from a simple precipitation. However, it was clearly demonstrated by the inverted light microscopy and time-lapse imaging that CPB are able to self-propagate during at least first 5 days of culture and probably even 20 days more^34^.

Increased serum calcium and phosphorus levels are associated with coronary artery disease, cerebrovascular events, peripheral artery disease, and heart valve calcification^35^,^36^,^37^,^38^,^39^,^40^,^41^,^42^,^43^,^44^,^45^,^46^,^47^,^48^,^49^. Furthermore, decreased serum fetuin-A and albumin levels are associated with the development of coronary artery disease and heart valve calcification^50^,^51^,^52^,^53^,^54^,^55^,^56^,^57^,^58^. Therefore, the risk factors of atherosclerosis and heart valve calcification are identical to those promoting the formation of CPB in the human blood. Hence, it raises a question about pathogenicity of CPB and their role in cardiovascular calcification. Conceivably, the pathogenic effects of CPB may be defined by at least three putative mechanisms: 1) endothelial toxicity; 2) direct tissue calcification; 3) functional changes in anti-calcification proteins impairing their function.

We found that CPB elicit endothelial toxicity which, in turn, defines their pathogenic effects since it is a crucial point in the development of atherosclerosis^59^,^60^. CPB promoted caspase-dependent apoptosis in endothelial cells, stimulated production of IL-6 and IL-8, which are established pro-atherosclerotic cytokines^61^,^62^, and induced intimal hyperplasia *in vivo*. These effects were caused by internalisation of CPB by endothelial cells. Our data correspond to the results from previous studies revealing a significant cytotoxicity of CPB for other cell lines^2^,^8^,^18^,^63^,^64^,^65^,^66^. Thus, CPB may act as an initial trigger of atherosclerosis via endothelial damage and may further enhance local inflammation through the induction of pro-atherosclerotic cytokines. Another possible mechanism might be a pro-atherosclerotic action of CPB through deregulation of HH pathway, previously reported to be inhibited in atherosclerotic plaques^31^,^32^.

To the best of our knowledge, we are the first who tested the ability of CPB to cause direct calcification both *in vitro* and *in vivo*. We could not observe pericardium calcification induced by CPB; however, the supersaturated solutions of CaCl_2_ and Na_2_HPO_4_*12H_2_O in equal concentrations of 1 mM or 10 mM did cause a significant calcification of pericardia. Therefore, we suggest that it is hypercalcemia/hyperphosphatemia that induces direct cardiovascular calcification, and CPB are not a culprit here. Moreover, CPB neutralise the deleterious effects of the supersaturation of the calcium salts and phosphates. This confirms the hypothesis of Wu, Young, and colleagues who proposed that CPB, which form in the biological fluids as a result of the supersaturation of calcium and phosphate ions, may be a part of a physiological cycle that regulates the function, transport and disposal of calcium and phosphorus in the human body^10^,^11^,^12^,^13^. Furthermore, we suggest that CPB which were found in the cardiovascular tissues by a number of research groups^21^,^22^,^23^,^24^,^25^,^26^,^27^,^28^ form there as a result of hypercalcemia/hyperphosphatemia and therefore should be considered as precursors but not inducers of calcification.

Finally, we investigated whether CPB can cause functional changes in anti-calcification proteins. We investigated BSA and HSF since these proteins represent two distinct mechanisms of calcium binding^4^. In HSF, surface binding of calcium is mediated by negative charges on the extended β-sheet of domain D1 that might occupy PO_4_ positions on the face of apatite crystals, leading to high affinity binding^4^. BSA binds calcium as counterions to numerous negatively charged amino acids facing the external milieu^4^. Therefore, HSF binds basic calcium phosphate with a high affinity whilst BSA binds free calcium with a low affinity^4^. We could not find any structural or functional changes in BSA and HSF as a result of the exposure to CPB; therefore, we suggest that CPB do not impair function of anti-calcification proteins.

In this study, we tested three potential pathogenic mechanisms of CPB. We demonstrated that it is apoptosis-mediated endothelial toxicity but not direct tissue calcification or functional changes in anti-calcification proteins that defines the pathogenic effects of CPB. Future studies on the pathogenicity of CPB should focus on their cytotoxic effects. It would be of interest to investigate endothelial toxicity in arterial endothelial cells for the better understanding of how CPB alter cell secretory profile and which apoptotic pathways they induce. This may help us to uncover mechanisms of atherosclerosis, particularly its initial trigger.

## Conclusion

Natural and artificial CPB are morphologically and chemically indistinguishable entities. Since the risk factors of atherosclerosis and the factors promoting the formation of CPB in the human blood are identical, it raises a question about their pathogenicity. We found that it is apoptosis-mediated endothelial toxicity but not direct tissue calcification or functional changes in anti-calcification proteins that defines the pathogenic effects of CPB. Future investigations should focus on the cytotoxicity of CPB, particularly alterations of a cell secretory profile. This may improve our understanding of atherosclerosis development.

## Experimental Section

### Isolation of calcium phosphate bions from calcified atherosclerotic plaques

The study was approved by the ethical committee of the Research Institute for Complex Issues of Cardiovascular Diseases (Kemerovo, Russian Federation), and a written informed consent was provided by all the patients after receiving a full explanation of the study. The segments of the calcified atherosclerotic plaques from aorta, carotid, or femoral artery of sixteen patients were explanted during the surgery. The tissue was homogenised and centrifuged at 3,000 × g for 10 min at 4 °C (in all experiments, for a low speed centrifugation we used 5804R Centrifuge, Eppendorf) to pellet the debris. Then, the supernatant was filtered through a cellulose acetate filter (Millipore) with 0.22 μm pore size. Finally, 3 mL of the filtrate were then aliquoted into a flask (T-25, Greiner) containing 7 mL of the culture medium (DMEM supplied with 10% FBS, 1% L-glutamine–penicillin–streptomycin solution, and 0.4% amphotericin B, Gibco). All procedures were performed under the sterile conditions. All culture of CPB was performed at 37 °C, 5% CO_2_, and high humidity (MCO-18AIC, Sanyo). The duration of culture was 6 weeks. The culture medium with and without the similar amount of phosphate buffered saline (PBS, 1x and pH 7.4, Gibco) and serum-free DMEM were used as a negative control. To ensure that the super-concentrated calcium salts and phosphates and not any other entities are the actual cause of the formation of CPB, we also performed the similar procedure with the samples of internal mammary artery. After 6 weeks of culture, the culture medium was centrifuged at 200,000 × g for 1 h at 4 °C (in all experiments, for a high speed centrifugation we used Optima MAX-XP Ultracentrifuge, Beckman Coulter). The pellet was dissolved in sterile PBS for the experiments on cells and animals or in sterile ddH_2_O for the investigation of the physicochemical properties. The quantification of CPB was performed by turbidimetry at a wavelength of 650 nm according to McFarland Latex standards (0.5, 1, and 2 McF with respective absorbance values of 0.08–0.10, 0.14–0.17, and 0.27–0.31). In all experiments, we used Uniplan microplate reader (Pikon) for the measurements of the OD.

### Artificial synthesis of calcium phosphate bions

For the artificial synthesis of CPB, we prepared the stock solutions of CaCl_2_ (0.45М, Sigma-Aldrich) and Na_2_HPO_4_*12H_2_O (0.2M, Sigma-Aldrich) and further diluted them to equal concentrations of 1 mM in a flask with 10 mL of the culture medium, incubated samples under the same conditions as above, and the same procedures were performed after 6 weeks of culture. For an artificial synthesis of CPB containing only BSA or HSF, we diluted the stock solutions of CaCl_2_ and Na_2_HPO_4_*12H_2_O in DMEM to equal concentrations of 1 mM, and then added either BSA (500 μg/mL, Sigma-Aldrich) or HSF (10 μg/mL, Sigma-Aldrich), respectively. All procedures were performed under the sterile conditions. The solutions were incubated for 3 days at 4 °C, centrifuged at 200,000 × g for 1 h at 4 °C, and finally dissolved in sterile ddH_2_O.

### Decalcification of calcium phosphate bions

For the decalcification of CPB, we centrifuged the CPB solution at 200,000 × g for 1 h at 4 °C and then treated the pellet with either 0.5M EDTA disodium salt dihydrate (C_10_H_14_N_2_Na_2_O_8_·2H_2_O, Sigma-Aldrich) at 4 °C constantly shaking overnight (O/N) or 0.5N HCl (Sigma-Aldrich) stirring for 5 min with a further neutralisation with an equal volume of 0.5N NaOH (Sigma-Aldrich).

### Cell culture

For the cell culture experiments, we used EA.hy 926 endothelial cells provided by Dr. Cora-Jean S. Edgell (University of North Carolina at Chapel Hill, USA), 2H-11 endothelial cells (ATCC), and a primary culture of human dermal fibroblasts (passage 5/6) isolated from a healthy adult leg skin. EA.hy 926 cell line was derived by fusing human umbilical vein endothelial cells with the human lung adenocarcinoma cell line A549 and retains the main morphological and functional features of human vein endothelial cells^67^. 2H-11 cell line was derived by transforming a primary culture of murine lymphoid endothelial cells with a simian virus 40 and also retains the main features of murine lymphoid endothelial cells^68^. Cells were cultured in DMEM/F12 (Sigma-Aldrich) supplemented with 10% fetal calf serum (Gibco), 2% hypoxanthine-aminopterin-thymidine (Sigma-Aldrich), 1% 4-(2-hydroxyethyl)-1-piperazineethanesulfonic acid (HEPES) buffer (Gibco), 1% L-glutamine–penicillin–streptomycin solution (Gibco), and 0.4% amphotericin B (Gibco). All the cell culture experiments were performed at 37 °C, 5% CO_2_, and high humidity (MCO-18AIC, Sanyo).

### Animals

Sixteen male Wistar rats weighing 250–300 g, 12–14 weeks of age were used for all animal experiments. The animals were allocated in the polypropylene cages (five animals per cage) lined with wood chips and had access to the water and food (rat chow) *ad libitum*. Throughout the whole time of experiment, the standard conditions of the temperature (24 ± 1 °C), relative humidity (55 ± 10%), and 12 h light/dark cycles were carefully maintained, and the health status of all rats was monitored daily. All procedures were in accordance with the *Guide for the Care and Use of Laboratory Animals*^69^ and were approved by the ethical committee of the Research Institute for Complex Issues of Cardiovascular Diseases.

### Electron and atomic force microscopy

Visualisation of CPB was performed using transmission electron microscopy (TEM), scanning electron microscopy (SEM), and atomic force microscopy (AFM). For TEM, we put a few drops of the CPB solution on a carbon-coated copper grid (Structure Probe, Inc.), stained the sample with 2% uranyl acetate (Electron Microscopy Sciences), and carried out TEM (JEOL JEM-2100, Jeol). For SEM, we pipetted a few drops of the CPB solution on a glass microscope slide (Thermo Scientific), dried at room temperature (RT) O/N, mounted the slides on a double sided adhesive conductive carbon tape (Ted Pella), sputter coated with Au-Pd (SC7640, Emitech), and finally performed SEM (Zeiss CrossBeam 1540 XB, Carl Zeiss). For AFM, we pipetted a few drops of the CPB solution on a mica disc (Ted Pella), and conducted AFM using Cypher™ Atomic Force Microscope (Asylum Research).

### Elemental analysis

To determine the chemical elements composing CPB, we pipetted a few drops of the CPB solution on a double sided adhesive conductive carbon tape, dried it for 2 h at 37 °C, sputter coated with carbon (CA7625, Emitech), and performed an elemental analysis by energy-dispersive X-ray spectroscopy (XFlash® 4010, Bruker). For each sample, we defined three quadrants where CPB were clearly observed, and then calculated the average atomic percent for each element. All calculations were performed after the carbon correction to avoid the misinterpretation of the result.

### X-ray diffraction analysis

A few drops of the CPB solution were deposited on a glass microscope slide and dried in a laminar flow hood at RT O/N. XRD spectra were obtained using a X-ray diffractometer (Bruker D8 ADVANCE, Bruker) with an X-ray ferrum tube operating at 40 kV. Data were collected over a 2θ angle ranging from 10 degrees to 100 degrees at a speed of 0.02 degree per second. Diffraction spectra were compared with the database of the Joint Committee on Powder Diffraction and Standards to identify the chemical formula of the crystalline compound.

### Fourier transform infrared spectroscopy

A few drops of the CPB solution were used as the samples for FTIR. FTIR spectra were acquired with a FTIR spectrometer (Infralum FT-801, Simex). The spectra were obtained at a resolution of 4 cm^−1^ and at wavelengths ranging between 4,000 cm^−1^ to 500 cm^−1^. Each spectrum represents an average of 25 consecutive scans.

### Dynamic light scattering measurements

A particle-size distribution curve of the CPB solution was calculated by DLS using Zetasizer Nano ZS (Malvern Instruments). The total volume of one sample was 1.5 mL, and for this experiment we passed ddH_2_O through the 0.22-μm cellulose acetate filter. Before the measurement, samples were incubated at 25 °C for 10 min. All measurements were performed thrice with the further calculation of the average distribution.

### Sodium dodecyl sulphate-polyacrylamide gel electrophoresis and silver staining

For the SDS-PAGE, equal aliquots (30 μL) of either intact CPB solution (0.5 McF) or CPB protein extract were mixed with Laemmli Buffer (1.5 M Tris-HCl pH 6.8, glycerol, β-mercaptoethanol, SDS, 1% bromophenol blue, dithiothreitol) at a ratio 4:1, and then loaded on 1.0 mm NuPAGE^®^ Novex^®^ 4–12% Bis-Tris protein gradient gel (Life Technologies). The Precision Plus protein standard (Bio-Rad) was loaded as a marker. Proteins were separated by the SDS-PAGE at 100 V for 1 h. The gel was stained using the Silver Stain Plus staining kit (Bio-Rad) according to the manufacturer’s instructions. Stop solution (39 mM EDTA disodium salt) was added as soon as bands started to appear. All gels were photographed using the HP Scanjet Enterprise Flow scanner (Hewlett Packard).

### Western blotting

For the immunoblotting, a small amount (10 μL) of the CPB solution (0.5 McF), was mixed with Laemmli Buffer at a ratio 4:1, and loaded on 1.0 mm NuPAGE® Novex® 4–12% Bis-Tris protein gradient gel. Normal human liver protein extract was used as a positive control. Proteins were separated by the SDS-PAGE at 100 V for 1 h and transferred at 30 V for 1.5 h 4 °C. Blots were probed with either fetuin-A (ab34505, Abcam) or albumin (ab9092, Abcam) antibodies. Horseradish peroxidase conjugated rabbit or goat secondary antibodies (Santa Cruz Biotechnology) were used at dilution 1:500. Proteins were visualised with enhanced chemiluminescence by using the Amersham ECL Prime detection reagent (General Electric Healthcare). For the experiments described in Figs 6e and 7a,b, total protein was extracted from cells using RIPA buffer (ThermoFisher) with protease inhibitor cocktail (Roche). Electrophoretically resolved protein samples were transferred and probed with cleaved caspase 9 (GTX86912, GenTex), xiap (sc-11426, Santa Cruz Biotechnology), htra2 (sc-15467, Santa Cruz Biotechnology), shh (sc-9024, Santa Cruz Biotechnology), ptch1 (sc-9016, Santa Cruz Biotechnology), and gapdh (sc-20357, Santa Cruz Biotechnology) antibodies.

### Nucleic acid staining

For the detection of the putative nucleic acids in CPB, we performed Hoechst 33342 staining. A few drops of the CPB solution were deposited on a glass microscope slide and dried in a laminar flow hood at RT for 30 min. Then, we pipetted a few drops of Hoechst 33342 (2 μg/mL, Sigma-Aldrich) onto dried CPB and incubated them at RT for 20 min. Visualisation was carried out by a fluorescent microscopy using AxioObserver.Z1 Inverted Microscope (Carl Zeiss) and Zeiss Filter Set #49 (ex. G 365 nm, FT 395, em. BP 445/50 nm). An empty slide stained with Hoechst 33342 was used as a negative control.

### DNA extraction and quantification

DNA isolation from CPB was carried out by phenol-chloroform method using a conventional protocol. Quantification of DNA was performed using NanoDrop 2000 UV-Vis Spectrophotometer (Thermo Scientific). Human leukocyte DNA and ddH_2_O were used as a positive and negative control, respectively.

### Lipid extraction and gas chromatography-mass spectrometry

Lipids were extracted from CPB according to a Folch method using a conventional protocol. GC-MS was performed using MDN-1 column (nonpolar methylsilicone, 30 m × 0.25 mm, Sigma-Aldrich) and GCMS-QP2010 Ultra (Shimadzu) according to the following parameters: injection volume 1 μL, injector temperature 200 °C, split ratio 1:10, interface temperature 210 °C, detector temperature 200 °C, carrier (He) flow rate 0.8 mL/min, temperature program 100°С for 2 min, 5°/min up to 120°С, 20°/min up to 260°С, then 260°С for 2 min. Mass range was 1.5-1,900 m/z.

### Dual inhibition-seeding assay

To estimate a dual inhibition-seeding ability of FBS, we incubated the sterile stock solutions of CaCl_2_ and Na_2_HPO_4_*12H_2_O diluted to equal concentrations of either 0.2, 0.5, 1, 5, or 10 mM in 1 mL of DMEM supplied with either 1, 3, 5, 7, 10, 15, or 20% FBS. To assess how the calcium salt/phosphate ratio may affect the formation of CPB, we incubated the sterile stock solutions of CaCl_2_ and Na_2_HPO_4_*12H_2_O diluted to either 4:1, 2:1, 1:1, 1:2, or 1:4 mM ratio in 1 mL of DMEM supplied with either 1, 3, 5, 7, 10, 15, or 20% FBS. The incubation was carried out in 1.66 mL tubes (Eppendorf) at 4 °C or in 24-well plates (Eppendorf) at 37 °C, 5% CO_2_, and high humidity (MCO-18AIC, Sanyo). All samples were plated in triplicate, and the average values were used for the further analysis. Sequential measurements of the OD at a wavelength of 650 nm were performed at the baseline, 1^st^, 3^rd^, 7^th^, 14^th^, 28^th^, and 42^nd^ day of the incubation.

### Self-propagation assay

To determine a self-propagation ability of CPB, we diluted 300 μl of the filtered calcified tissue homogenate or the sterile stock solutions of CaCl_2_ and Na_2_HPO_4_*12H_2_O to equal concentrations of 1 mM in 1 mL of the culture medium. The incubation was carried out in 1.66 mL tubes at 4 °C. All samples were plated in triplicate, and the average values were used for the further analysis. An assessment of a self-propagation ability was performed by sequential measurements of the OD at a wavelength of 650 nm at the baseline, 1^st^, 3^rd^, 7^th^, 14^th^, 28^th^, and 42^nd^ day of the incubation. For time-resolved DLS analysis of the CPB solution (Fig. 5e,f), we diluted 300 μl of the filtered calcified tissue homogenate in 1 mL of the culture medium and then performed sequential DLS measurements at the baseline, 1^st^, and 7^th^ day of the incubation.

### Internalisation assay

EA.hy 926 cells in the 6-well plates (2*10^5^ cells per well) were exposed to 100 μl of either CPB (2 McF) or PBS for 48 h. The cell pellet was fixed for 1 h with 2.5% glutaraldehyde (Sigma-Aldrich), postfixed for 1 h with 1% osmium tetroxide (Serva Electrophoresis), embedded into 2% agarose (Amresco), and dehydrated in a graded ethanol series. The sections of the agarose gel were embedded in a 1:1 (w/w) epoxy resin:acetone (Sigma-Aldrich) mixture at RT for 2 h, then embedded again in an epoxy resin at RT O/N, and finally embedded in a fresh epoxy resin. Ultrafine sections were performed using an ultramicrotome (LKB Bromma Nova Ultra Microtome, LKB), placed on a carbon-coated copper grid, stained with a 2% uranyl acetate, and counterstained with a lead citrate (Electron Microscopy Sciences). The sections were then examined with a transmission electron microscope (JEOL JEM-2100, Jeol).

### Annexin V/7-aminoactinomycin D assay

EA.hy 926 cells and primary human dermal fibroblasts in the 6-well plates (2*10^5^ cells per well) were exposed to 100 μl of either CPB (0.5 or 2 McF) or sterile PBS for 24 h. Annexin V/7-aminoactinomycin D assay for the detection of apoptosis/necrosis was performed with the respective kit of BioLegend according to the manufacturer’s instructions, and the results were measured using flow cytometry (BD FACSCalibur Flow Cytometry System, BD Biosciences). Analysis of the flow cytometry data was carried out by FCAP Array software (Soft Flow). All samples were measured in duplicate, and the average concentrations were used for the further analysis.

### 3-(4,5-dimethylthiazol-2-yl)-2,5-diphenyltetrazolium bromide assay

EA.hy 926 cells and primary human dermal fibroblasts in the 96-well plates (2*10^4^ cells per well) were exposed to 10 μl of either CPB (0.5 or 2 McF) or sterile PBS for 24 h. MTT (3-(4,5-dimethylthiazol-2-yl)-2,5-diphenyltetrazolium bromide) assay for the assessment of cell viability and proliferation was conducted with the respective kit of Life Technologies according to the manufacturer’s instructions, and the results were measured at a wavelength of 570 nm.

### Enzyme-linked immunosorbent assay

EA.hy 926 cells and primary human dermal fibroblasts in the 6-well plates (2*10^5^ cells per well) were exposed to 100 μl of either CPB (0.5 or 2 McF) or sterile PBS for 24 h. The cytokine and cell adhesion molecule (CAM) levels (IL-1α, -4, -6, -8, sCD40L, GM-CSF, sE-selectin, sP-selectin, sICAM-1, IFN-γ, and TNF-α) were measured in a cell culture supernatant by ELISA using the respective kits of eBioscience according to the manufacturer’s instructions. All samples were plated in duplicate, and the average concentrations were used for the further analysis. The results were measured at a wavelength of 450 nm.

### Animal model

After the induction of anesthesia with a 3% isoflurane, all animals received inhalation anesthesia with a 1.5% isoflurane during the whole time of surgery. We used the original experimental model of rat aorta angioplasty with a coronary angioplasty balloon catheter^70^. Briefly, aorta was punctured in the proximal direction with a 21 gauge needle, DIOR 2.0 × 15 mm balloon catheter was then inserted with a 0.014 inch guideware into the aortic lumen, and an angioplasty was finally carried out with inflation pressure 10 atm for 10 sec. The catheter was withdrawn and advanced thrice in order to denude the endothelium. All procedures were performed using strict aseptic technique. All rats were divided into the sample and control groups with the equal number of animals in each (7 rats). Therefore, while still under anesthesia, either CPB solution (700 μL, 0.5 McF) or equal volume of the physiological saline (Gibco) was inoculated into the rat bloodstream via tail vein injection.

### Tissue collection, staining, and histological analysis

Five weeks postoperation, all rats were sacrificed by an overdose of carbon dioxide. Segments of rat abdominal aortas (≥2.5 cm) were removed, fixed in 10% (w/v) neutral phosphate buffered formalin (Electron Microscopy Sciences), embedded in paraffin (Electron Microscopy Sciences), sectioned (3 μm), and mounted on the glass microscope slides. Alternatively, for alizarin red S staining, snap-frozen tissue blocks were cut on a cryostat (Microm HM 525, Thermo Scientific), and sections were mounted on the glass microscope slides. After hematoxylin and eosin (H&E), van Gieson, von Kossa, and alizarin red S staining according to the conventional protocols, adjacent sections from each aorta were evaluated by light microscopy (Axioscop 40 Lab Microscope, Carl Zeiss) in a blinded fashion for the extent of intimal hyperplasia based on IMR and for the calcium deposits. Calcified bioprosthetic heart valve was used as a positive control. Intimal and medial areas were calculated using ImageJ (National Institutes of Health). Three sections per stain were assessed from each rat, and the mean values were estimated for the statistical analysis.

### *In vitro* calcification model

The pieces of the commercially available bovine and porcine pericardia (NeoCor) fixed in 0.625% glutaraldehyde for 1 month were incubated in the culture medium at 37 °C, 5% CO_2_, and high humidity (MCO-18AIC, Sanyo). The incubation was carried out in the 6-well plates, four pericardium pieces per well. Samples were divided into four groups, exposed either to the CPB solution (200 μL, 2 McF), to CaCl_2_ and Na_2_HPO_4_*12H_2_O in equal concentrations of 1 mM or 10 mM, or to 200 μL of sterile PBS. All samples were plated in duplicate. The histological examination of the distinct pieces from the same well was performed at the 3^rd^ and 6^th^ week of the incubation. Tissue samples were cut on a cryostat as described above, stained with alizarin red S, and finally evaluated (AxioImager.A1, Carl Zeiss) in a blinded fashion for the calcium deposits. Calcified bioprosthetic heart valve was used as a positive control.

### Preparation of the samples for circular dichroism and calcification propensity assay

BSA (45 mL, at 50 mg/mL in sterile ddH_2_O) was incubated with 5 mL of either BSA-CPB solution (2 McF) or sterile ddH_2_O. The mixture was placed in Falcon tubes (Greiner) at 37 °C, 5% CO_2_, and high humidity (MCO-18AIC, Sanyo) for 1 week. HSF (9 μl, at 1 μg/μl in sterile ddH_2_O) was incubated with 1 μl of either HSF-CPB solution (2 McF) or sterile ddH_2_O. The mixture was placed in 1.66 mL tubes at 37 °C, 5% CO_2_, and high humidity (MCO-18AIC, Sanyo) for 1 week. Then, all solutions were frozen at −40 °C O/N and further lyophilised O/N (FreeZone Plus 2.5 Liter Cascade Benchtop Freeze Dry System, Labconco). After the lyophilisation, approximately a half of the powder from all samples underwent decalcification by 0.5M EDTA disodium salt dihydrate for 6 h at RT, was again frozen at −40 °C O/N, and was further lyophilised O/N. All samples were prepared in duplicate.

### Circular dichroism

Spectra were recorded at 20 °C on a Jasco J-600 (Jasco) dichrograph using 1 mm thick quartz cells. Spectra of BSA were measured between 185 and 270 nm with a scanning speed of 50 nm/min, resolution and band width of 1 nm, and sensitivity of 50 mdeg. All the settings for the measurement of HSF spectra were the same except the sensitivity of 5 mdeg. Each spectrum represents an average of 3 and 5 consecutive scans for BSA and HSF, respectively. The concentrations of BSA and HSF were 0.01 μg/μL and 0.055 μg/μL, respectively.

### Precipitation assay

Native BSA (45 mL, at 50 mg/mL in sterile physiological saline) was incubated with 5 mL of either BSA-CPB solution (2 McF) or sterile physiological saline. The mixture was placed in Falcon tubes at 37 °C, 5% CO_2_, and high humidity (MCO-18AIC, Sanyo) for 1 week. The concentration of soluble protein was checked at the baseline, 1^st^, 3^rd^, 5^th^, and 7^th^ day using a Bradford assay (GENESYS 6 UV-Vis spectrophotometer, Thermo Scientific) according to the conventional protocol. All samples were plated in duplicate, and the average values were used for the further analysis.

### Calcification propensity assay

Lyophilised BSA incubated with either BSA-CPB solution (2 McF) or sterile ddH_2_O with or without further decalcification was dissolved in DMEM up to a concentration of 500 μg/mL, and the sterile stock solutions of CaCl_2_ and Na_2_HPO_4_*12H_2_O were then diluted in this solution to equal concentrations of 0.1, 0.2, 0.5, 1, and 2 mM. The similar procedure was performed with lyophilised HSF (10 μg/mL). After 48 h of incubation at 4 °C, we measured the OD at a wavelength of 650 nm to assess an anti-calcification capacity of treated and untreated BSA and HSF. All samples were plated in duplicate, and the average values were used for the further analysis.

### Statistical analysis

Statistical analysis was performed using GraphPad Prism (GraphPad Software). A sampling distribution was assessed by D’Agostino-Pearson test and Kolmogorov-Smirnov test. Regarding descriptive statistics, data were represented by the mean and range. Two independent groups were compared by two-tailed Student’s t-test. An adjustment for multiple comparisons was performed using false discovery rate (FDR). P-values, or q-values if FDR was applied (q-values are the name given to the adjusted p-values found using an optimised FDR approach), ≤0.05 were regarded as statistically significant.

## Additional Information

**How to cite this article**: Kutikhin, A. G. *et al*. Apoptosis-mediated endothelial toxicity but not direct calcification or functional changes in anti-calcification proteins defines pathogenic effects of calcium phosphate bions. *Sci. Rep.* **6**, 27255; doi: 10.1038/srep27255 (2016).
